# American Mistletoe as an Oxytocic and Emmenagogue

**Published:** 1888-06

**Authors:** J. M. G. Carter

**Affiliations:** Waukegan, Illinois


### THE AMERICAN MISTLETOE AS AN OXYTOCIC AND EMMENAGOGUE.

BY J. M. G. CARTER, M.D., Sc.D., Ph.D.
Waukegan, Illinois.

The American mistletoe, *Phoradendron flavescens* (Nutt.), *Viscum flavescens* (Pursh.), belongs to the *Loranthaceæ*, or mistletoe family, of which there are five or six species in the United States. This, and some other species of America, are sometimes called false mistletoe.

The American mistletoe is an evergreen, shrubby plant, one to three feet high, growing as a parasite on the branches of various trees, chiefly on the black gum and red elm. The brittle stems are jointed and much-branched, with a stiff, leathery or parchment-like foliage of a greenish or yellowish-green color. The leaves are thick, firm, and persistent, abovate or oval in shape, somewhat petioled, longer than the spikes in their axils. Exceptionally, only scales appear in place of leaves.

In the Southern States it flowers in April and May. In California, where there are two varieties of this species, it is said to flower in February and March. The flower is fragrant, having the odor of pond-lilies. Notes on the evolution of the bud and natural history of this plant were kindly furnished me by my friend, Dr. J. Schneck, of Mt. Carmel, Illinois, who is one of the most practical botanists in the State. They were published in the *Botanical Gazette* in 1884.

I am not aware of any experiments made to determine the physiological action of this important drug, but it appears, from clinical observations, to have some decided control over the capillary circulation in general, but especially in the neighborhood of the genito-urinary organs. Indirectly, it seems to have some influence over mucous membrane, particularly in the bladder and urethra. But that the effect upon the capillary circulation is general, is shown in part by the fact that both vesical and cerebral inflammations and conjestions are subdued by the drug.

Dr. W. H. Long considers the mistletoe to be a better remedy, as an oxytocic, than ergot, and claims that it acts more promptly and surely; he says it is an excellent remedy in any form of uterine hæmorrhage, especially in post-partum cases. In cases of labor it should be given in the form of fluid extract, the dose being one drachm every twenty minutes. I would prescribe it under the same precautions as ergot. In menorrhagia the fluid extract may be given in drachm doses four to six times a day.

The value of this drug was first brought to my attention by Dr. J. Schneck. His attention was drawn to it by the use made of it by the "old women" in domestic practice. In the southern part of this State, and perhaps in other localities where it grows in abundance, it is freely used in domestic practice in the form of "tea" for girls who have taken cold and are suffering with suppression of the menses. Dr. Schneck's trial of it justified the common practice. He recommended the plant to me, and my experiments with it corroborated his testimony in its favor. The "tea," or decoction, may be used *ad libitum.*

I used it in one case of orchitis, where there was irritable urethra, and was pleased with its effect. I did not succeed in securing a fluid extract of this drug until three years ago. Since then I have used it constantly in cases of irritable urethra, in cystitis, in congestions and inflammations of the urino-genital organs resulting from exposure to cold, and in those cases of dysmenorrhœa and menorrhagia due to some derangement of the local circulation. I have been so well pleased with the results following its use that I have seldom thought it necessary to change the treatment. I have tried this remedy in congestive neuralgias of the ovaries and uterus, but the results have not been sufficiently marked to encourage me to recommend the drug in these cases.

